# Bias and Missing Data: Representation of Older Adults With Sensory Impairment in Epidemiologic Studies

**DOI:** 10.1093/geroni/igaa057.2994

**Published:** 2020-12-16

**Authors:** Bonnielin Swenor, Alden Gross

## Abstract

Diverse research populations are necessary to maximize the generalizability of results. Differences between people, including age, gender, racial or ethnic origin may impact observed associations in epidemiologic studies or influence the efficacy and safety of interventions in controlled trials. For these reasons, the diversity of research study populations is critical to public health and well-being. While there have been concerted efforts to examine and enhance the representation of older adults in research studies, not all subgroups of these populations, including those with sensory impairments, have been considered. Approximately 55% of adults 60 years and older have a vision and/or hearing impairment, yet despite this high prevalence, little attention has been paid to determining if or how sensory impairment affects research study participation or measurement of outcomes. This symposium aims to address these gaps, with a focus on how vision and hearing impairments may be associated with bias and missing data in epidemiologic studies of cognition and cognitive aging. The objective of this session is to highlight ongoing work in this novel area, and spark discussion and collaborations to catalyze this area of research.

